# Factors Associated With Postpartum Uninsurance Among Medicaid-Paid Births

**DOI:** 10.1001/jamahealthforum.2021.1054

**Published:** 2021-06-14

**Authors:** Jamie R. Daw, Katy B. Kozhimannil, Lindsay K. Admon

**Affiliations:** 1 Department of Health Policy and Management, Columbia Mailman School of Public Health, New York, New York; 2 Division of Health Policy and Management, University of Minnesota School of Public Health, Minneapolis; 3 Department of Obstetrics and Gynecology, University of Michigan, Ann Arbor; 4 Institute for Healthcare Policy and Innovation, University of Michigan, Ann Arbor

## Abstract

**Importance:**

To reduce postpartum uninsurance and improve postpartum health, the American Rescue Plan included an option for states to extend pregnancy-related Medicaid from 60 days to 1 year after childbirth. Recent estimates of postpartum uninsurance among Medicaid-paid births would provide information on who would benefit from state adoption of this extension.

**Objective:**

To estimate rates of postpartum uninsurance among individuals with Medicaid-paid births.

**Design, Setting, and Participants:**

This cross-sectional study used survey data from the 2015 to 2018 Pregnancy Risk Assessment Monitoring System (PRAMS), a representative sample of births in 43 states and New York City, New York, and included PRAMS participants for whom Medicaid was the primary payer for childbirth. The mean weighted PRAMS response rate was 60.9% for the sites and years included in this study. The data were analyzed from December 2020 to January 2021.

**Interventions or Exposures:**

Self-reported postpartum uninsurance measured at the time of the PRAMS survey (mean [interquartile range], 4.2 (3.0-5.0) months after birth).

**Main Outcomes and Measures:**

Survey-weighted rates of postpartum uninsurance and 95% CIs overall, by state, by state Medicaid expansion status, and by maternal sociodemographic characteristics. Adjusted odds ratios of the association between maternal characteristics and postpartum uninsurance.

**Results:**

We identified 63 370 respondents with a Medicaid-paid birth. Of these, 22 016 (41.1%) were non-Hispanic White individuals, 17 442 (22.0%) non-Hispanic Black individuals, 6808 (13.3%) Spanish-speaking Hispanic individuals, 7000 (13.7%) English-speaking Hispanic individuals, 2410 (4.0%) Asian/Pacific Islander individuals, and 3894 (1.3%) Indigenous individuals. Of these, 41.7% were no longer insured by Medicaid postpartum and 22.0% were uninsured postpartum. The postpartum uninsurance rate was 3 times higher in Medicaid nonexpansion states (36.8%) compared with expansion states (12.8%). Postpartum uninsurance varied significantly across states, ranging from 1.7% in Massachusetts to 56.7% in Texas. There were substantial racial and ethnic disparities in postpartum uninsurance: 54.9% of Hispanic, Spanish-speaking individuals (adjusted odds ratio [aOR], 6.2; 95% CI, 5.5-7.0) and 42.8% of Indigenous respondents reported postpartum uninsurance (aOR, 4.3; 95% CI, 3.8-4.9) compared with 15.2% of non-Hispanic White respondents. The odds of postpartum uninsurance was higher among unmarried people (aOR, 1.3 compared with married people; 95% CI, 1.2-1.4), those 35 years and older (aOR, 1.5 compared with those younger than 20 years; 95% CI, 1.2-1.9) and those with lower levels of education (aOR, 0.8 for more than high school compared with less than high school; 95% CI, 0.7-0.9).

**Conclusions and Relevance:**

In this cross-sectional survey study of 43 states, a high proportion of people with Medicaid-paid births were uninsured in the postpartum period, particularly those living in Medicaid nonexpansion states. The study findings suggest that state extensions of pregnancy-related Medicaid eligibility through the first year postpartum could disproportionately benefit Hispanic and Indigenous people, unmarried people, those with lower education levels, and those living in Medicaid nonexpansion states.

## Key Points

**Question:**

What factors are associated with postpartum uninsurance among Medicaid-paid births?

**Findings:**

In this cross-sectional survey study of 63 370 respondents with a Medicaid-paid birth in 43 states, 41.7% were no longer insured by Medicaid postpartum and 22.0% were uninsured. Postpartum uninsurance after a Medicaid-paid birth varied widely by state, Medicaid expansion status, and maternal race/ethnicity.

**Meaning:**

State extensions of pregnancy-related Medicaid eligibility through the first year postpartum may to disproportionately benefit Hispanic and Indigenous people and those living in Medicaid nonexpansion states.

## Teaser

This cross-sectional survey examines rates of postpartum uninsurance among individuals with Medicaid Medicaid-paid births.

## Introduction

During the weeks and months following birth, many postpartum individuals experience physical, emotional, and mental health challenges.^1^ Further, half of all pregnancy-related deaths happen after birth, and a third happen beyond the first week postpartum.^2^ Health insurance facilitates financial access to timely health care services to mitigate maternal morbidity and mortality, treat acute postpartum conditions, and manage ongoing chronic conditions after birth.

The Medicaid program plays an important role in supporting postpartum health. Medicaid pays for nearly half of all US births and an even greater proportion of births among low-income people and people of color, who bear a disproportionate share of maternal morbidity and mortality.^3,4^ In states that report pregnancy-related deaths by insurance status, most are among women with Medicaid coverage (for example, 62% in Louisiana, 69% in Texas, and 83% in West Virginia).^5,6,7^

Federal law requires all states to provide Medicaid coverage to low-income pregnant individuals with incomes up to 138% of the federal poverty level (FPL) ($29 974 for a family of 3), although many states have extended eligibility to higher income levels (median, 200% of the FPL or $43 440 for a family of 3).^8^ However, pregnancy-related Medicaid coverage is time limited from the beginning of pregnancy to 60 days after birth. In many states, particularly in the 12 states that have not adopted the US Affordable Care Act’s state Medicaid expansion for low-income adults, there is a large gap in income eligibility for pregnant individuals and low-income parents or adults.^9^ For example, in Texas, pregnant people qualify for Medicaid at incomes up to 207% of the FPL, but only parents making 17% of the FPL ($3692 annually for a family of 3) remain eligible for Medicaid at 60 days postpartum.^8,10^

Over the past year, multiple states have introduced or passed legislation to extend pregnancy-related Medicaid to 1 year postpartum, and several states have Medicaid 1115 waivers pending to do so.^11^ In March 2021, Congress passed the American Rescue Plan (ARP), which included an option for states to extend postpartum Medicaid for 1 year with federal matching funds.^12^ This legislation allows states to bypass the 1115 waiver process and implement an extension through a state plan amendment. As of April 15, 2021, no states have announced that they will adopt the option in the ARP, although on April 12, 2021, the US Centers for Medicare & Medicaid Services approved the first Medicaid 1115 waiver to extend postpartum coverage in Illinois.

We use survey data from multiple states to describe postpartum uninsurance for individuals with Medicaid-paid births overall, by state, by state Medicaid expansion status, and by maternal sociodemographic characteristics. By providing timely data on the prevalence of postpartum uninsurance in each state and the characteristics of Medicaid beneficiaries who are most likely to be affected, this analysis can inform state decisions to adopt a postpartum Medicaid extension.

## Methods

### Data

In this cross-sectional analysis, we used the 2015 to 2018 Pregnancy Risk Assessment Monitoring System (PRAMS) survey data collected in 43 states and New York City, New York. The US Centers for Disease Control and Prevention (CDC) Division of Reproductive Health administers PRAMS in collaboration with state health departments.^13^ From birth certificate data, participating states select a representative sample of live births each month. Postpartum participants complete a standardized mail or telephone survey. Data include demographic characteristics, insurance status, health care utilization, and health outcomes before, during, and after birth. For each survey year, the CDC only reports data from states that meet minimum response rate thresholds (50% in 2018; 55% from 2015-2018). The data were deidentified, and this study was deemed exempt from review by the University of Michigan institutional review board. This study follows Strengthening the Reporting of Observational Studies in Epidemiology (STROBE) reporting guidelines for cross-sectional studies.

### Sample

We identified PRAMS respondents with a live birth from 2015 to 2018 for whom Medicaid was the primary payer for birth. To identify the primary payer for birth, we used 2 insurance variables that are provided in the PRAMS data: (1) the primary payer recorded by the delivery institution (eg, hospital) on the birth certificate (96.8% of respondents), and if that was missing, (2) self-reported payer for birth (0.3% of respondents). For the 11.5% of respondents with both of these variables, responses were 87.7% consistent. We excluded respondents who were missing both variables (2.9% of the PRAMS sample) because we were unable to assess their eligibility for inclusion.

### Variables

Our primary outcome was postpartum uninsurance based on responses provided at the time of the postpartum survey, which 97.4% of respondents completed 3 or more months after childbirth (mean [interquartile range], 4.2 (3.0-5.0 months). The PRAMS respondents were able to select more than 1 source of insurance coverage. Following previous studies of perinatal insurance using PRAMS data,^14,15,16^ we hierarchically characterized insurance coverage into 1 of 3 mutually exclusive categories: Medicaid, private, or uninsured. The Medicaid category included respondents who reported enrollment in Medicaid or a state-named Medicaid program. The private category included those who reported private insurance alone, in combination with Medicaid, TRICARE, or other military insurance. A few respondents (5.2%) reported having private insurance and Medicaid coverage (and were classified as privately insured). The uninsured category included respondents who reported having no insurance. Consistent with the US Census,^17^ other national surveys,^18^ and previous analyses of PRAMS,^14^ women who reported only Indian Health Service (IHS) were also classified as uninsured.

To classify race/ethnicity, we used variables provided in PRAMS that are derived from the birth certificate. These self-reported variables are typically collected using a maternal form and entered into the birth certificate by the facility where the birth occurred. The PRAMS does not include information on maternal place of birth or immigration status; however, language is included and is one of the most frequently used acculturation.^19^ Language has been used as a proxy for nativity or acculturation in other studies of health disparities among Hispanic populations in the US.^20^ Combining the information on race/ethnicity and survey language, we defined 7 mutually exclusive race/ethnicity categories: White non-Hispanic, Black non-Hispanic, Spanish-speaking Hispanic, English-speaking Hispanic, Indigenous (American Indian or Alaskan Native), Asian and Pacific Islander, and Other/mixed race.

Other covariates included household income as a percentage of the FPL, marital status, maternal education, parity, and self-reported indicators of ever receiving a diagnosis of diabetes, hypertension, or depression before pregnancy. US Affordable Care Act Medicaid expansion status was assigned at the state-year level, indicating whether Medicaid was expanded in a given state and year of birth.^21^ States with Medicaid expansions that occurred after June (midpoint of the year) were not considered expansion states until the following year. For example, individuals in Alaska (expansion on September 1, 2015) would be in the Medicaid nonexpansion group in 2015 and the Medicaid expansion group in years 2016 to 2018 (eTable 1 in the Supplement).

### Statistical Analysis

We calculated rates of postpartum uninsurance among Medicaid-paid births overall and by maternal characteristics. To measure the association between postpartum uninsurance and maternal characteristics, we estimated odds ratios (ORs) using multivariate logistic regression models. Given the wide differences in Medicaid eligibility for low-income adults/parents in Medicaid expansion and nonexpansion states,^9^ we presented all analyses overall and stratified by state Medicaid expansion status. In secondary analyses, we also calculated postpartum uninsurance rates by state. All analyses applied the survey weights provided by the CDC to account for the complex survey design.^13^ Individuals with missing covariate information for covariates were included in the regression model using a separate missing category indicator. We used 2-sided statistical tests to compare estimates of interest using a significance level of .05. All analyses were conducted in Stata, version 15.1 (StataCorp).

## Results

The mean weighted PRAMS response rate was 60.9% for the sites and years included in this study. The response rates for each state and year are reported in eTable 2 in the Supplement. We identified 64 887 respondents with a Medicaid-paid delivery from January 2015 to December 2018. We excluded 1217 individuals (1.9%) with missing data for the primary outcome, resulting in a final sample size of 63 370 individuals with Medicaid-paid births. Table 1 shows the characteristics of the study sample overall and by state Medicaid expansion status. Most of the sample was aged 20 to 29 years with high school or higher education levels and incomes less than 138% of the FPL. Compared with expansion states, more individuals in nonexpansion states were younger than 25 years, non-Hispanic Black, nulliparous, had an income less than 100% of the FPL, and had hypertension.

**Table 1.** Sample Characteristics

Characteristic	No. (%)^a^		
	All Medicaid-paid births (n = 63 670)	Medicaid expansion states (n = 41 061)	Medicaid nonexpansion states (n = 21 022)
Age, y			
<20	5700 (9.0)	3294 (7.6)	2406 (11.3)^b^
20-24	17 829 (28.8)	10 762 (26.6)	7067 (32.5)^b^
25-29	19 438 (30.5)	12 613 (31.1)	6825 (29.5)^b^
30-34	12 974 (19.8)	8855 (21.1)	4119 (17.8)^b^
≥35	7729 (11.8)	5538 (13.7)	2191 (8.9)^b^
Education level			
Less than HS	14 467 (22.7)	9094 (22.3)	5373 (23.3)
High school	23 497 (37.9)	14 606 (36.6)	8891 (40.1)^b^
More than HS	25 032 (38.5)	16 846 (40.0)	8186 (36.0)^b^
Missing	674 (0.9)	515 (1.1)	159 (0.6)^b^
Marital status			
Married	41 543 (63.9)	26 282 (62.8)	15 261 (65.6)^b^
Missing	95 (0.1)	66 (0.1)	29 (0.1)
Income, % of the FPL			
<100%	26 173 (39.6)	16 111 (37.6)	10 062 (42.7)^b^
100%-138%	13 420 (21.0)	8349 (20.5)	5071 (21.9)^b^
139%-199%	8468 (13.7)	5596 (13.6)	2872 (13.8)
≥200%	6960 (11.3)	4871 (12.0)	2089 (10.3)^b^
Missing	8649 (14.4)	6134 (16.3)	2515 (11.3)^b^
Race/ethnicity			
Non-Hispanic			
White	22 016 (41.1)	14 416 (41.7)	7600 (40.1)^b^
Black	17 442 (22.0)	10 506 (20.6)	6936 (24.2)^b^
Hispanic			
Spanish	6808 (13.3)	4706 (13.1)	2102 (13.6)
English	7000 (13.7)	4792 (13.3)	2208 (14.4)
Asian/Pacific islander	2410 (4.0)	1838 (5.0)	572 (2.3)^b^
Indigenous	3894 (1.3)	2270 (1.2)	1624 (1.6)^b^
Other/mixed^c^	3391 (3.4)	1984 (3.6)	1407 (3.1)^b^
Missing	709 (1.1)	549 (1.4)	160 (0.6)^b^
Parity			
Nulliparous	21 305 (33.6)	13 750 (32.9)	7555 (34.7)^b^
1 previous live birth	18 466 (30.7)	12 103 (31.1)	6363 (30.0)
≥2 live births	23 771 (35.6)	15 112 (35.9)	8659 (35.2)
Missing	128 (0.2)	96 (0.2)	32 (0.1)
Chronic conditions			
Diabetes	2485 (3.7)	1579 (3.7)	906 (3.6)
Missing	1069 (1.6)	714 (1.9)	355 (1.2)^b^
Hypertension	4724 (6.3)	2833 (5.9)	1891 (6.9)^b^
Missing	935 (1.4)	612 (1.6)	323 (1.1)^b^
Depression	11 098 (16.0)	6937 (16.2)	4161 (15.8)
Missing	900 (1.3)	606 (1.5)	294 (0.9)^b^

Abbreviations: FPL, federal poverty level; HS, high school.

^a^ Estimates are unweighted sample sizes and survey-weighted percentages

^b^ Difference between expansion and nonexpansion states is statistically significant at the .05 level.

^c^ Other/mixed race/ethnicity category includes “Other Non-White” and “Mixed Race.”

**Figure 1.**
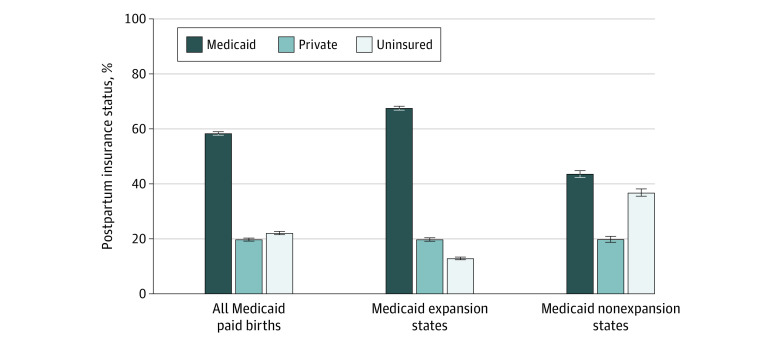
Postpartum Insurance Status for Medicaid-Paid Births by State Medicaid Expansion Status Estimates are survey-weighted percentages and 95% CIs.

Figure 1 displays the postpartum insurance status for Medicaid-paid births overall and by state Medicaid expansion status. Overall, only 58.3% of individuals with a Medicaid-paid birth were still insured by Medicaid postpartum. Twenty two percent reported being uninsured postpartum and 19.7% reported private insurance. The postpartum uninsurance rate was 2.5 times higher in nonexpansion states compared with expansion states (36.8% vs 12.8%).

Table 2 shows the rates of postpartum uninsurance by maternal characteristics overall and by Medicaid expansion status. Overall, uninsurance rates were higher than average among unmarried individuals (26.1%), those 35 years and older (25.8%) and those with less than a high school education (29.8%). More than half (54.9%) of Hispanic, Spanish-speaking individuals, 27.5% of Hispanic, English-speaking individuals, and 42.8% of Indigenous individuals reported postpartum uninsurance compared with 15.2% of White non-Hispanic individuals. For all race/ethnicity groups, postpartum uninsurance rates were significantly higher in Medicaid nonexpansion states compared with expansion states.

**Table 2.** Postpartum Uninsurance by Maternal Characteristics

Characteristic	% (95% CI)		
	All Medicaid-paid births	Medicaid expansion states	Medicaid nonexpansion states^a^
Overall	22.0 (21.4-22.6)	12.8 (12.4-13.3)	36.8 (35.5-38.1)
Age, y			
<20	15.0 (13.2-17.1)	7.9 (6.6-9.4)	22.8 (19.4-26.5)
20-24	21.6 (20.4-22.9)	10.6 (9.7-11.5)	36.1 (33.8-38.5)
25-29	21.9 (20.8-23.0)	12.5 (11.7-13.3)	37.8 (35.5-40.1)
30-34	23.7 (22.4-25.1)	14.9 (13.9-16.1)	40.3 (37.3-43.4)
≥35	25.8 (24.0-27.8)	17.5 (16.0-19.0)	46.4 (41.7-51.2)
Education level			
Less than HS	29.8 (28.3-31.3)	20.3 (19.1-21.5)	44.4 (41.5-47.3)
High school	20.7 (19.7-21.7)	11.5 (10.8-12.3)	34.0 (31.9-36.1)
More than HS	18.8 (17.9-19.7)	9.6 (9.0-10.3)	35.0 (33.0-37.1)
Marital status			
Unmarried	26.1 (25.1-27.2)	16.3 (15.6-17.2)	42.9 (40.6-45.2)
Married	19.8 (19.0-20.5)	10.7 (10.2-11.3)	33.6 (32.0-35.2)
Income, % of the FPL			
<100%	22.9 (21.9-24.0)	11.9 (11.2-12.7)	38.5 (36.5-40.5)
100%-138%	21.6 (20.2-23.1)	11.3 (10.4-12.4)	37.0 (34.2-39.8)
139%-199%	21.7 (20.1-23.5)	13.3 (12.1-14.7)	35.0 (31.5-38.7)
≥200%	17.9 (16.3-19.5)	12.5 (11.2-13.9)	27.9 (24.4-31.8)
Race/ethnicity			
Non-Hispanic			
White	15.2 (14.4-16.0)	6.8 (6.2-7.4)	29.0 (27.3-30.8)
Black	12.1 (11.2-13.1)	5.8 (5.2-6.6)	20.7 (18.8-22.8)
Hispanic			
Spanish	54.9 (52.8-57.0)	43.3 (41.3-45.2)	72.9 (68.6-76.8)
English	27.5 (25.4-29.7)	12.5 (11.3-13.9)	49.5 (45.1-53.8)
Asian/Pacific islander	17.3 (14.8-20.1)	11.8 (10.0-13.9)	36.3 (28.0-45.5)
Indigenous	42.8 (40.2-45.5)	28.8 (26.3-31.4)	60.2 (55.0-65.2)
Other/mixed^b^	17.6 (15.5-19.9)	9.4 (7.4-11.7)	32.8 (28.2-37.7)
Parity			
Nulliparous	19.5 (18.5-20.6)	11.6 (10.8-12.4)	31.5 (29.4-33.8)
1 previous live birth	22.2 (21.1-23.3)	13.1 (12.3-14.0)	37.2 (34.9-39.6)
≥2 live births	24.3 (23.2-25.4)	13.7 (12.9-14.5)	41.6 (39.4-43.9)
Chronic conditions			
Diabetes	18.9 (16.2-21.9)	11.7 (9.4-14.3)	30.9 (25.0-37.5)
Hypertension	17.8 (15.7-20.2)	8.9 (7.3-10.8)	30.1 (25.8-34.7)
Depression	14.8 (13.6-16.1)	7.1 (6.2-8.2)	27.4 (24.8-30.2)

Abbreviations: FPL, federal poverty level; HS, high school.

^a^ Overall and for all characteristics, the difference between expansion and nonexpansion states is statistically significant at the .05 level.

^b^ Other/mixed race/ethnicity category includes “Other Non-White” and “Mixed Race.” Missing categories not shown.

**Figure 2.**
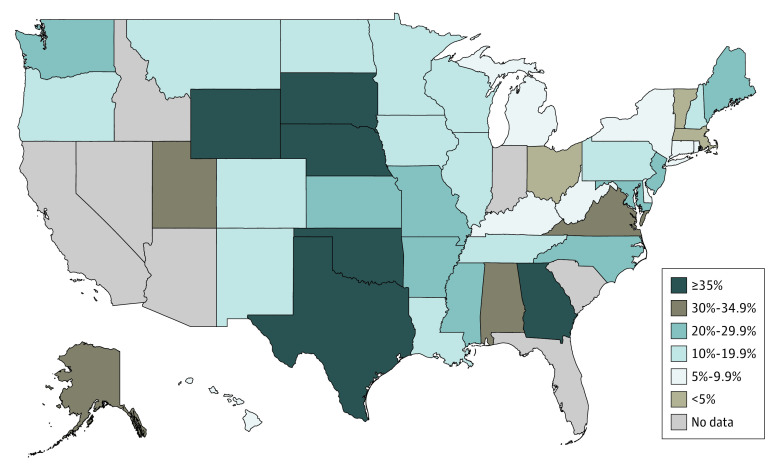
Postpartum Uninsurance Rates Among Medicaid-Paid Births by State Estimates are survey-weighted percentages.

There was wide state variation in the postpartum uninsurance rate among Medicaid-paid births (Figure 2; eTable 3 in the Supplement). Among states that had implemented the Medicaid expansion in all years of the study period, the uninsurance rate varied from 1.7% in Massachusetts to 21.9% in New Jersey. Among Medicaid nonexpansion states, the uninsurance rate varied from 9.7% in Tennessee to 56.7% in Texas. Three states had uninsurance rates less than 5%: Massachusetts (1.7%), Vermont (3.5%), and Ohio (4.4%). Six states had uninsurance rates higher than 35%: Georgia (36.2%), Nebraska (37.1%), Wyoming (41.6%), South Dakota (46.3%), Oklahoma (51.4%), and Texas (56.7%).

Table 3 shows the adjusted ORs (aORs) from the logistic regression models overall and stratified by state Medicaid expansion status. eTable 4 in the Supplement presents marginal differences in the predicted probability of uninsurance based on the adjusted logistic regression model. In adjusted regression analyses, individuals younger than 20 years were significantly less likely to be uninsured postpartum compared with all other age groups. The age gradient was larger in Medicaid nonexpansion states, where the odds of uninsurance was 3 times higher among individuals 35 years and older compared with those younger than 20 years (aOR, 2.5; 95% CI, 1.8-3.6). Individuals with more than high school education had lower odds of postpartum uninsurance compared with those with less than high school education overall (aOR, 0.8; 95% CI, 0.7-0.9) and in expansion states (aOR, 0.7; 95% CI, 0.6-0.7), but this association was not significant in nonexpansion states. Married individuals had lower odds of postpartum uninsurance compared with unmarried individuals (aOR, 0.8; 95% CI, 0.7-0.9). Overall, household income was not associated with postpartum uninsurance. However, within expansion states, incomes greater than 138% of the FPL had significantly higher odds of uninsurance compared with individuals with incomes less than 100% of the FPL (aOR for ≥200% of the FPL, 1.6; 95% CI, 1.3-1.9). In nonexpansion states, individuals with higher incomes (≥200% of the FPL) had significantly lower odds of postpartum uninsurance compared with individuals with incomes less than 100% of the FPL (aOR, 0.7; 95% CI, 0.6-0.9).

**Table 3.** Predictors of Postpartum Uninsurance

Characteristic	aOR (95% CI)		
	All Medicaid-paid births	Medicaid expansion states	Medicaid nonexpansion states
Age, y			
<20	1 [Reference]	1 [Reference]	1 [Reference]
20-24	1.6 (1.4-2.0)^a^	1.5 (1.2-1.9)^a^	2.2 (1.7-2.8)^a^
25-29	1.6 (1.3-2.0)^a^	1.8 (1.4-2.3)^a^	2.4 (1.8-3.1)^a^
30-34	1.6 (1.3-1.9)^a^	2.0 (1.5-2.5)^a^	2.3 (1.7-3.1)^a^
≥35	1.5 (1.2-1.9)^a^	2.0 (1.5-2.7)^a^	2.5 (1.8-3.6)^a^
Education level			
Less than HS	1 [Reference]	1 [Reference]	1 [Reference]
High school	0.9 (0.8-1.0)^a^	0.8 (0.7-0.9)^a^	0.9 (0.7-1.0)
More than HS	0.8 (0.7-0.9)^a^	0.7 (0.6-0.7)^a^	1.0 (0.8-1.2)
Marital status			
Unmarried	1.3 (1.2-1.4)^a^	1.5 (1.3-1.6)^a^	1.1 (0.9-1.3)
Income, % of the FPL			
<100%	1 [Reference]	1 [Reference]	1 [Reference]
100%-138%	1.0 (0.9-1.2)	1.1 (1.0-1.3)	1.0 (0.9-1.2)
139%-199%	1.1 (0.9-1.2)	1.4 (1.2-1.6)^a^	0.9 (0.8-1.1)
≥200%	0.9 (0.8-1.1)	1.6 (1.3-1.9)^a^	0.7 (0.6-0.9)^a^
Race/ethnicity			
Non-Hispanic			
White	1 [Reference]	1 [Reference]	1 [Reference]
Black	0.8 (0.7-0.9)^a^	0.9 (0.8-1.1)	0.6 (0.5-0.7)^a^
Hispanic			
Spanish	6.2 (5.5-7.0)^a^	9.2 (8.0-10.4)^a^	6.0 (4.7-7.6)^a^
English	2.1 (1.9-2.4)^a^	2.0 (1.7-2.3)^a^	2.5 (2.1-3.1)^a^
Asian/Pacific islander	1.0 (0.8-1.3)	1.4 (1.1-1.8)^a^	1.3 (0.9-1.9)
Indigenous	4.3 (3.8-4.9)^a^	6.4 (5.4-7.4)^a^	3.8 (3.0-4.9)^a^
Other/mixed^b^	1.2 (1.0-1.4)^a^	1.4 (1.1-1.9)^a^	1.3 (1.0-1.6)
Parity			
Nulliparous	1 [Reference]	1 [Reference]	1 [Reference]
1 previous live birth	1.0 (0.9-1.1)	0.9 (0.8-1.0)	1.1 (0.9-1.3)
≥2 live births	1.0 (0.9-1.2)	0.8 (0.7-0.9)^a^	1.1 (0.9-1.3)
Chronic conditions			
Diabetes	0.8 (0.5-1.1)	1.0 (0.7-1.3)	0.8 (0.5-1.1)
Hypertension	0.9 (0.7-1.2)	0.9 (0.7-1.1)	0.9 (0.7-1.2)
Depression	0.8 (0.7-0.9)^a^	0.8 (0.7-0.9)^a^	0.8 (0.7-0.9)^a^

Abbreviations: aOR, adjusted odds ratio; FPL, federal poverty line; HS, high school.

^a^ Comparison with the reference category is statistically significant at the .05 level.

^b^ Other/mixed race/ethnicity category includes “Other Non-White” and “Mixed Race.” Missing categories not shown.

We observed significant associations between race/ethnicity and postpartum uninsurance. The adjusted odds of postpartum uninsurance were 6.2 (95% CI, 5.5-7.0) and 2.1 (95% CI, 1.9-2.4) times higher among Hispanic, Spanish-speaking and Hispanic, English-speaking individuals, respectively, compared with White non-Hispanic individuals. This translates to a difference of 37.2 percentage points (95% CI, 34.6-39.7) in the adjusted probability of postpartum uninsurance for Hispanic, Spanish-speaking individuals compared with non-Hispanic White individuals (eTable 4 in the Supplement). For most groups, the magnitude of the associations between race/ethnicity and postpartum uninsurance were larger in Medicaid expansion states; for example, Hispanic, Spanish-speaking individuals were more likely to be uninsured postpartum compared with non-Hispanic White individuals in expansion states vs nonexpansion states (aOR, 9.2; 95% CI, 8.0-10.4 vs aOR, 6.0; 95% CI, 4.7-7.6). Indigenous individuals also had higher odds of postpartum uninsurance compared with White non-Hispanic individuals in expansion states (aOR, 6.4; 95% CI, 5.4-7.4) vs nonexpansion states (aOR, 3.8; 95% CI, 3.0-4.9). The aORs were not significant for diabetes, hypertension, or parity.

## Discussion

Using survey data from 43 states from 2015 to 2018, we found that 22% of individuals with a Medicaid-paid birth were uninsured postpartum. The rate of postpartum uninsurance was 3 times higher in states that did not expand Medicaid under the Affordable Care Act (36.8%) compared with Medicaid expansion states (12.8%), likely reflecting greater access to Medicaid for low-income adults regardless of pregnancy status. Rates of postpartum uninsurance ranged across states from 1.7% in Massachusetts to 56.7% in Texas. Several groups were particularly vulnerable to uninsurance during the postpartum period, including Hispanic and Indigenous individuals and those who were unmarried, 35 years and older, and with less than high school education.

The rates of postpartum uninsurance were markedly high among people of color in Medicaid nonexpansion states. Most notably, 72.9% of Hispanic, Spanish-speaking, 49.5% of Hispanic, English-speaking, and 60.2% of Indigenous individuals did not have insurance postpartum in nonexpansion states. The high rate of uninsurance among Hispanic, Spanish-speaking women is consistent with prior research and likely reflects limited health insurance options for immigrant people.^16^ Many states do not allow undocumented people and recent immigrants (within 5 years) to enroll in traditional Medicaid programs; thus, many Medicaid-paid births among this group are likely covered by emergency Medicaid (which only covers the childbirth hospitalization) or the unborn child option. There is an urgent need to address coverage gaps for all pregnant people in the US, particularly as research has found higher rates of pregnancy complications and postpartum depression among undocumented people.^22^

Indigenous people, particularly Indigenous rural residents, are at increased risk for severe maternal morbidity and mortality and may face unique barriers to care.^23,24^ For example, relationships created by US treaty obligations may enable enrolled tribal members to access health care via the IHS, but do not always provide health insurance coverage for other health care clinicians or facilities.^25^ Many IHS facilities do not provide prenatal or childbirth services, which may require Indigenous people to use the same type of emergency Medicaid coverage for childbirth hospitalization that is frequently required for immigrant individuals who do not have other health insurance options.^26^

Notably, while the overall rates of uninsurance were considerably lower, the racial/ethnic disparities in postpartum uninsurance compared with White non-Hispanic individuals were still large in expansion states, reflecting structural inequities that persist even in states with more generous eligibility guidelines. This highlights that extending postpartum Medicaid alone will not be enough to address disparities in postpartum uninsurance by race and ethnicity, particularly if extensions do not provide options for pregnant and postpartum immigrants or attention to the specific needs of Indigenous people.

The association between household income and postpartum insurance differed by state Medicaid expansion status, reflecting different Medicaid eligibility criteria in Medicaid expansion and nonexpansion states. In expansion states, only women earning up to 138% of the FPL continued to be eligible for Medicaid after pregnancy-related Medicaid ends. As a result, we found higher odds of postpartum uninsurance among women earning more than 138% of the FPL. In nonexpansion states, the median Medicaid eligibility threshold for parents was 40% as of January 2020.^10^ As a result, the uninsurance rates were more similar between income groups (as all comprise women who lose eligibility), and only the highest income group (≥200% of the FPL) had a lower odds of postpartum uninsurance, likely reflecting an increased uptake of private insurance. While the ARP extension option requires full extension of benefits for all incomes that quality for pregnancy-related Medicaid in a given state, it is unclear whether some states may try to pursue a 1115 waiver with more limited income eligibility (or if the Centers for Medicare & Medicaid Services would approve these waivers). Although people with incomes between 100% and 400% of the FPL have access to subsidies to purchase Marketplace coverage (which have become more generous within the ARP), we find that the transition to Marketplace coverage is not seamless, and individuals in this income range also experience high rates of uninsurance in expansion and nonexpansion states. This may reflect affordability issues that persist even after subsidies and difficulties in navigating insurance enrollment during the already challenging postpartum period. The results of this study suggest that postpartum Medicaid extensions that cover all incomes that qualify for pregnancy Medicaid, including those eligible for Marketplace subsidies, would have the greatest association with reducing postpartum uninsurance.

This study expands on other research that documents high rates of insurance changes and uninsurance in the postpartum period among births covered by all payers in the US.^16,27,28^ By focusing specifically on Medicaid beneficiaries, this analysis describes the characteristics of individuals who would be most likely to benefit in states that adopt a Medicaid extension through 1 year postpartum under the ARP state plan amendment or through a similar 1115 waiver. It also identifies states that have particularly high levels of postpartum uninsurance (eg, Texas, Oklahoma, and South Dakota) and thus may see particularly large gains in insurance if an extension is adopted. Assuming all states adopted a postpartum Medicaid extension at their state income eligibility level for pregnancy Medicaid, up to 12.8% of Medicaid-paid births (58 827) in expansion states and 36.8% of Medicaid-paid births (501 244) in nonexpansion states would have the option for continued Medicaid coverage. These numbers are likely overestimates, because some Medicaid-paid births that were followed by uninsurance may have been among eligible individuals and emergency Medicaid and the unborn child option would not be subject to an extension under current federal or state proposals.

### Limitations

This study has limitations that should be considered when interpreting the results. First, the PRAMS postpartum insurance variable is self-reported and thus may be subject to measurement error. Second, while the PRAMS sample includes many states with different geographies and demographic characteristics, the results should not be considered nationally representative estimates and may not be generalizable to jurisdictions that did not participate in PRAMS during the study period. The 7 excluded states (Arizona, California, Florida, Idaho, Indiana, Nevada, and South Carolina) and Washington DC represented approximately 27.6% of Medicaid paid births in the US in 2018.^29^ Third, we are unable to discern immigration status, and thus eligibility, for emergency Medicaid vs pregnancy-related Medicaid. Fourth, while all included states met the CDC PRAMS response rate thresholds, the average state response rate varied (from 55.4% in Texas to 69.6% in New Jersey), as did the number of years a state was included in the sample (mean [range], 3 [1-4] years) (eTable 2 in the Supplement). This resulted in reduced precision of state-level estimates for states with lower response rates and/or fewer years of inclusion (eTable 3 in the Supplement).

## Conclusions

In this cross-sectional survey study of 43 states, we found that many people with Medicaid-paid births were uninsured in the postpartum period, particularly those living in Medicaid nonexpansion states. If state postpartum Medicaid extensions were to be implemented for all people with a Medicaid paid birth, the results of this study suggest that these extensions would disproportionately benefit Hispanic and Indigenous people, unmarried people, those with lower education levels, and those living in Medicaid nonexpansion states.

## Notes

Supplement. eTable 1. Included PRAMS sites by Medicaid expansion status eTable 2. PRAMS Weighted Response Rates, by PRAMS Site and Year eTable 3. Postpartum Uninsurance Rates Among Medicaid Paid Births, by PRAMS site eTable 4. Adjusted Differences in the Predicted Probability of Uninsurance
