# Supplementary material for: Factors Associated With Postpartum Uninsurance Among Medicaid-Paid Births
Source: JAMA Health Forum. 2021 Jun 14;2(6):e211054. doi: 10.1001/jamahealthforum.2021.1054 (PMC8796978; doi:10.1001/jamahealthforum.2021.1054)
Supplement: Supplement. — eTable 1. Included PRAMS sites by Medicaid expansion status eTable 2. PRAMS Weighted Response Rates, by PRAMS Site and Year eTable 3. Postpartum Uninsurance Rates Among Medicaid Paid Births, by PRAMS site eTable 4. Adjusted Differences in the Predicted Probability of Uninsurance [file jamahealthforum-e211054-s001.pdf]

Supplemental Online Content

Daw JR, Kozhimannil KB, Admon LK. Factors associated with postpartum uninsurance among Medicaid-paid births. *JAMA Health Forum*. 2021;2(6):e211054. doi:10.1001/jamahealthforum/2021/1054

eTable 1. Included PRAMS sites by Medicaid expansion status
eTable 2. PRAMS Weighted Response Rates, by PRAMS Site and Year
eTable 3. Postpartum Uninsurance Rates Among Medicaid Paid Births, by PRAMS site
eTable 4. Adjusted Differences in the Predicted Probability of Uninsurance

This supplemental material has been provided by the authors to give readers additional information about their work.

© 2021 Daw JR et al. *JAMA Health Forum.*

eTable 1. Included PRAMS sites by Medicaid expansion status

Medicaid Expansion	Medicaid Nonexpansion
Alaska (2016-18)	Alaska (2015)
Arkansas	Alabama
Colorado	Georgia
Connecticut	Kansas
Delaware	Louisiana (2015-16)
Hawaii	Maine
Illinois	Mississippi
Iowa	Missouri
Kentucky	Nebraska
Louisiana (2017-18)	North Carolina
Massachusetts	Oklahoma
Maryland	South Dakota
Michigan	Tennessee
Minnesota	Texas
Montana	Utah
North Dakota	Virginia
New Hampshire	Wisconsin
New Jersey	Wyoming
New Mexico	
New York	
New York City	
Ohio	
Oregon	
Pennsylvania	
Rhode Island	
Vermont	
Washington	
West Virginia	

© 2021 Daw JR et al. *JAMA Health Forum.*

eTable 2. PRAMS Weighted Response Rates, by PRAMS Site and Year

PRAMS Site	Response Rate				Total No. of Years	Mean Weighted Response Rate (%)
	2015	2016	2017	2018		
Alabama	59.6		55.3	54.2	3	56.4
Alaska	66.2	61.3	61.9	60.2	4	62.4
Arkansas	62.6	55.8		52	3	56.8
Colorado	63.3	59.3	62.5	61.6	4	61.7
Connecticut	61.5	59.7	60.5	58.4	4	60.0
Delaware	63.9	63.7	61.8	59.4	4	62.2
Georgia			68.3	59.2	2	63.8
Hawaii	61.8	56.3			2	59.1
Illinois	66.3	59.9	54.6	61.4	4	60.6
Iowa	62.7	62.9	62.2		3	62.6
Kansas			62.9	60.8	2	61.9
Kentucky			59.4	60.2	2	59.8
Louisiana	65.8	63.4	66	64.3	4	64.9
Maine	57.6	55.9	61.9	57.3	4	58.2
Maryland	65.1	61.9	57.2		3	61.4
Massachusetts	62.5	59.9	61.9	62.4	4	61.7
Michigan	55.3	54.7	56.1	57.7	4	56.0
Minnesota				56.8	1	56.8
Mississippi				60.2	1	60.2
Missouri	66.2	62.3	63.6	56.8	4	62.2
Montana			61.4		1	61.4
Nebraska	62.8	60		60.6	3	61.1
New Hampshire	62	60.3	55.4	53.1	4	57.7
New Jersey	70.2	70.7	70	67.3	4	69.6
New Mexico	63.5	63.7	63.8	59.8	4	62.7
New York	62.7	65.8	56.4	50.8	4	58.9
New York City	72.4	72.5	67.2	64.7	4	69.2
North Carolina			56.5		1	56.5
North Dakota			70.2	59.9	2	65.1
Ohio	57.5				1	57.5
Oklahoma	67.9	63.1	59.7		3	63.6
Oregon	56.3				1	56.3
Pennsylvania	68.8	63.5	63.8	61.2	4	64.3
Rhode Island		59.5	58.6	57.6	3	58.6

© 2021 Daw JR et al. *JAMA Health Forum.*

South Dakota			66.9	64.3	2	65.6
Tennessee	60.2				1	60.2
Texas	56.2	54.6			2	55.4
Utah	67.1	64.9	66.3	62.2	4	65.1
Vermont	69.7	70.5	68.2	68.8	4	69.3
Virginia	63.7	60.4	56.5	63.2	4	61.0
Washington	59.1	58.2	61.4	61.5	4	60.1
West Virginia	59.2	57.3	55.1	55.6	4	56.8
Wisconsin	59	56.3	58.9	55	4	57.3
Wyoming	55.6	62.7	58.7	61.8	4	59.7
**Total No. of States**	33 + NYC	30 + NYC	34 + NYC	39 + NYC	**Mean =** 3 years	**Mean =** 60.9%
**Mean Weighted Response Rate (%)**	62.8	61.3	61.5	59.7		

Note: The CDC weighted response rate threshold for reporting PRAMS data was 50% in 2018 and 55% from 2015-2017. States must meet this threshold to have data released to researchers. Response rate information obtained from the CDC: https://www.cdc.gov/prams/prams-data/researchers.htm

© 2021 Daw JR et al. *JAMA Health Forum.*

eTable 3. Postpartum Uninsurance Rates Among Medicaid Paid Births, by PRAMS site

State	Postpartum Uninsurance Rate, Pct (95% CI)	State	Postpartum Uninsurance Rate, Pct (95% CI)
AK	31.6 (29.4, 33.9)	ND	15.8 (12.1, 20.5)
AL	29.9 (26.8, 33.2)	NE	37.1 (34.1, 40.2)
AR	21.0 (17.0, 25.7)	NH	13.1 (9.6, 17.5)
CO	18.7 (16.6, 21.0)	NJ	21.9 (19.8, 24.2)
CT	8.6 (7.3, 10.2)	NM	11.6 (10.5, 12.8)
DE	8.4 (7.1, 10.0)	NY	8.2 (6.2, 10.9)
GA	36.2 (31.9, 40.8)	OH	4.4 (2.3, 8.3)
HI	7.4 (4.5, 12.1)	OK	51.4 (48.7, 54.1)
IA	10.4 (8.5, 12.7)	OR	10.7 (8.3, 13.6)
IL	17.0 (15.3, 18.9)	PA	10.5 (8.7, 12.7)
KS	25.8 (21.3, 31.0)	RI	7.9 (6.5, 9.5)
KY	6.3 (4.3, 9.2)	SD	46.3 (42.3, 50.4)
LA	14.9 (13.4, 16.5)	TN	9.7 (6.7, 13.8)
MA	1.7 (1.2, 2.5)	TX	56.7 (53.4, 59.9)
MD	21.2 (19.0, 23.6)	UT	33.4 (30.7, 36.3)
ME	21.3 (18.6, 24.1)	VA	31.4 (26.7, 36.6)
MI	7.8 (6.5, 9.2)	VT	3.5 (2.6, 4.7)
MN	13.0 (9.1, 18.3)	WA	21.2 (19.2, 23.2)
MO	21.8 (19.5, 24.3)	WI	15.2 (13.4, 17.1)
MS	22.6 (19.0, 26.5)	WV	7.3 (5.9, 8.9)
MT	15.8 (13.1, 19.0)	WY	41.6 (37.4, 46.0)
NC	20.1 (15.9, 25.1)	NYC	10.5 (9.4, 11.7)

© 2021 Daw JR et al. *JAMA Health Forum.*

eTable 4. Adjusted Differences in the Predicted Probability of Uninsurance

	All Medicaid Paid Births	Medicaid Expansion States	Medicaid Nonexpansion States
**Age**			
<20	Ref	Ref	Ref
20-24	6.9 (4.6, 9.2)*	3.2 (1.5, 5.0)*	14.0 (9.8, 18.2)*
25-29	6.7 (4.3, 9.1)*	4.7 (2.9, 6.6)*	15.8 (11.2, 20.3)*
30-34	6.2 (3.6, 8.8)*	5.7 (3.8, 7.7)*	14.9 (9.9, 19.9)*
>=35	5.8 (3.0, 8.6)*	6.1 (3.9, 8.2)*	17.1 (10.8, 23.4)*
**Education**			
Less than HS	Ref	Ref	Ref
High School	-2.0 (-3.7, -0.3)*	-2.1 (-3.4, -0.9)*	-2.8 (-6.3, 0.7)
More than HS	-2.8 (-4.6, -1.0)*	-4.1 (-5.4, -2.8)*	-0.5 (-4.2, 3.3)
**Marital Status**			
Unmarried	3.6 (2.3, 4.9)*	3.8 (2.8, 4.8)*	2.1 (-4.9, 0.6)
**Income, % FPL**			
<100%	Ref	Ref	Ref
100-138%	0.6 (-1.1, 2.3)	0.9 (-0.3, 2.1)	0.7 (-2.6, 4.0)
139-199%	0.8 (-1.2, 2.7)	3.3 (1.8, 4.8)*	-1.7 (-5.7, 2.3)
>=200%	-1.1 (-3.2, 0.9)	4.6 (2.8, 6.4)*	-7.2 (-11.3, -3.1)*
**Race-Ethnicity**			
Non-Hispanic White	Ref	Ref	Ref
Non-Hispanic Black	-2.8 (-4.1, -1.4)*	-0.6 (-1.6, 0.4)	-8.6 (-11.4, -5.9)*
Hispanic, Spanish	37.2 (34.6, 39.7)*	32.6 (30.4, 34.9)*	40.9 (35.9, 45.9)*
Hispanic, English	12.4 (10.1, 14.7)*	5.9 (4.4, 7.3)*	21.5 (16.9, 26.2)*
Asian Pacific islander	0.5 (-2.2, 3.1)	2.6 (0.8, 4.3)*	5.6 (-2.8, 14.0)
Indigenous	28.1 (25.2, 30.9)*	24.5 (21.8, 27.2)*	31.3 (25.7, 36.9)*
Other/Mixed	2.7 (0.3, 5.0)*	2.7 (0.5, 4.9)*	4.7 (-0.3, 9.8)
**Parity**			
Nulliparous	Ref	Ref	Ref
1 previous live birth	0.1 (-1.4, 1.7)	-0.9 (-2.2, 0.4)	1.4 (-1.8, 4.6)
>=2 live births	0.5 (-1.3, 2.3)	-2.1 (-3.4, -0.8)*	2.1 (-1.6, 5.7)
**Chronic Conditions**			
Diabetes	-2.3 (-5.4, 0.8)	-0.1 (-2.9, 2.6)	-5.2 (-11.8, 1.5)
Hypertension	0.2 (-2.5, 2.9)	-1.1 (-3.4, 1.1)	-1.4 (-6.3, 3.5)
Depression	-3.9 (-5.5, -2.3)*	-2.2 (-3.7, -0.8)*	-5.0 (-8.1, -1.9)*

Notes: Estimates are differences in predicted probabilities and 95% confidence intervals relative to the reference group calculated from an adjusted survey weighted logistic regression at observed sample

© 2021 Daw JR et al. *JAMA Health Forum.*

values. Other/Mixed race-ethnicity category includes “Other Non-White” and “Mixed Race”. Missing categories not shown. *Comparison to reference category is statistically significant at the 0.05 level.

© 2021 Daw JR et al. *JAMA Health Forum.*
